# Her Heart's Mistake

**Published:** 1889-05-11

**Authors:** E. D. Cumming

**Affiliations:** Author of "An Ordeal by Silence"


### Her Heart's Mistake.

By E. D. Cumming, Author of "An Ordeal by Silence."

*(Continued from page 77.)*

### Chapter V. "Yes."

"Oh, by the way, Kate, Captain Bairdsley is coming in to breakfast this morning," said Mrs. Brent to her niece. "I had a long talk with him at the club last night. I must say that he is exceedingly agreeable; I like him much better than I did."

Miss Harrison did not answer. She had suddenly become deeply interested in the eccentric behaviour of a squirrel, which was looking to see how much water there was in the well in the corner of the compound.

"He is a better stamp of man than one generally sees out here," continued Mrs. Brent, taking a seat near the rocking-chair her niece occupied; "but men do lose polish sadly in this country if they stay long."

"I daresay that men think the same of us," answered Miss Harrison absently.

"I'm afraid that the conduct of many women justifies the opinion," said Mrs. Brent; "look at Mrs. Macdonald, for instance."

Mrs. Macdonald, the Collector's wife, was a homely, outspoken person of middle age, who made no attempt to live up to the position her husband's official rank would have entitled her to assume as head of Meerut society. She had been nearly twenty years in India, and long residence in isolated district stations, where she had no one of her own sex to speak to for months at a stretch, had had its effect upon her. She was neglectful in matters of dress, embarrassingly blunt in speech, and her little mannerisms supplied her neighbours with many anecdotes. She spoke the language like a native, and could drive a better bargain with a "box-wallah," or travelling pedlar, than any sergeant's wife in the lines. India was her world, the Viceroy her sovereign. She had been twice to England with her husband and did not like the country, whose social restraints galled her after the freedom of the East.

"I wasn't good enough for the folks at home," she was wont to say, after she returned the last time; "and I'm not going back again if I can help it. When Macdonald retires, we'll settle down at Ooty. I couldn't put up with English ways and English servants, and I know when I'm well off. Khitmugar! bring me a whisky-and-soda, and make haste."

But in spite of her peculiarities Mrs. Macdonald had many friends; and Miss Harrison, who could see below the rugged surface and appreciate the sound intrinsic qualities it concealed, was one of the staunchest.

"I am very fond of Mrs. Macdonald," said Kate, with a smile, as the short, stout figure, with its dishevelled hair, clad in its favourite garb—a very old and dingy cashmere dressing-gown—rose to her mind's eye. "She is a rough diamond, but if I were in trouble I'd rather go to her than any woman I know. What time did you tell Captain Bairdsley to come to breakfast, Aunt Agnes?" she enquired, after a pause.

"Half-past nine; and he is sure to be punctual."

Miss Harrison knew that was a certainty, and she retired from the verandah, where she had spent the early morning, to her room; whence she reissued half an hour later in all the freshness of white muslin.

"I am sorry that you were unwell last evening," said Captain Bairdsley, as he stepped on to the verandah. "I had some news for you."

"I hope it hasn't taken wings during the night," laughed Kate. "Were you able to keep it for me? What is it?"

"We have organised a sky meeting for next Thursday, and I got the committee to put a hurdle-race on the programme."

"Why did you want a hurdle-race?"

"Didn't you want one for your beloved Baroness?"

"Yes; and you did it for me. It *was* good of you to remember it," said Kate, looking up at him.

"I was sure you would like to have an opportunity of seeing what she could do; so as I was asked to act as a steward, I made a point of having a hurdle-race on the card. Some of the fellows wanted a steeplechase, but as that would not have suited Baroness, it didn't suit me."

"You *are* good," said Kate again. "You will ride her for me, of course."

"Will you entrust the honour of your colours to me?" he asked.

"I wouldn't let anyone else ride her, so you *must*," said Kate imperiously. "What colours will you wear?"

"I think I may safely leave that to you, but you must decide at once, because the entries are to be in by to-morrow morning. I have got a list of the races with me," he added, drawing a paper from his pocket.

Bairdsley took his seat beside her on a long cane lounge, and spread the paper out upon his knee. The two heads were very close together as the owners bent over the document, and it is possible that to this accident was due the fact that Mrs. Brent, coming out of her room on to the verandah, stepped quietly back again without disturbing them.

"Now, which is our race?" enquired Kate, passing a slender finger down the list.

"Our race?" said George Bairdsley, with slight but lingering emphasis on the possessive pronoun. "This is it—the fourth: the hurdle race, Rs.150 (that's the prize we are going to win), for all horses which have never won a race of the value of Rs.200 or upwards; weight for age and class; distance about one mile, over six flights of hurdles, about 3ft. high; entrance, Rs.5. To start at 5.30 p.m. It will be the best race of the day—at least the fellows thought so last night," said Bairdsley.

"Well, will you do everything for me? Write to Mr. Strachan—if he is the secretary this time—and tell him that Baroness is going to run, and that you are going to ride him?"

"Very well, I'll send in your entry, but what about colours? We must declare them."

"What shall we have?"

"What are you going to wear that day?"

"Oh, I don't know; I've got a very pretty dress of cream, with dark blue, that will do very well."

"Then, may I wear the same? Cream jacket, with dark blue sleeves and belt."

Kate accepted the suggestion with a flush of pleasure.

"Yes, I should like that," she said. "Have you got the jacket ready?"

"If you will leave the matter to me I'll get it made at once."

And this important affair having been settled, the two went into the dining-room, where Dr. Harrison and Mrs. Brent were already seated at the breakfast-table.

Neither Captain Bairdsley nor Kate had much to say that morning. The Doctor's inexhaustible fund of anecdote was taxed to its utmost to keep matters lively, though when breakfast was over, and the latter had been charged by her father to take care of their friend and amuse him, she accepted the task readily enough.

"What shall we do?" she enquired, as they went into the drawing-room together. "Shall we have some music?"

"Presently," answered Bairdsley, "just now I want to talk to you."

His tone and manner betrayed what he wanted to say, and Kate turned from the music-stand, to face him with heightened colour.

"What is it, Captain Bairdsley?" she asked.

"This, Kate. That I love you. Nothing more. What more need I say? I have loved you since the first day I saw you, and now I cannot live without you. Do you love me? Answer me, Kate!"

He came a step nearer her, and she grew white, almost terrified by the fierce impetuosity with which he spoke. His voice was low and harsh, and there was a look in his eyes that she had never seen in any man's before. She was taken aback by the suddeness of his declaration, which gave her no time to collect her thoughts.

"I have known you scarcely two months," she murmured; "how can I give you an answer?"

Bairdsley's face grew dark, and he seized her wrist as he spoke again.

"And is not two months long enough? Have we not been together day after day? Do we not know each other as well as though our lives had been passed under the same roof? Are you going to tell me now that you like me as a friend, and nothing more? Have you been leading me on to love you to cast me away when your sport was done?"

"No, no," cried Kate, trembling with agitation and distress; "you must not think that; I ——"

But he did not allow her to finish; he caught her in his arms and stopped the words with passionate kisses, heedless of her efforts to release herself. "Tell me that you love me, my darling," he whispered hoarsely.

She knew not what to answer; only a few days ago she had lain thinking of him, and telling herself that she did love him, and nursing jealously the thought that he loved her. Why could she not give him the reply he pressed for now? She tried gently to disengage herself from his arms, but he would not let her go.

"I will have an answer," he said firmly; "if you tell me that you do not love me, I will never look upon your face again. Answer me, Kate; I will go if you bid me, and never return again."

She could not tell him to go. She knew that she looked daily for his coming, and that her world would be a blank without him. She did love him, she was sure, but why could she not give him his answer?

"Give me time to think," she cried imploringly. "I cannot tell you to go away. Do not force me to answer you now."

He released her, and stood with folded arms, looking into her eyes.

"Either you love me or you do not, Kate," he said. "Which is it?"

She sank into a chair and pressed her hands to her face.

"Am I to go?" he asked after a minute's silence.

She looked up at him with tearful eyes, but still she could not bring herself to give the answer that would detain him. She had to choose at once; either she must accept his love or lose him altogether. She clenched her hands tightly as she thought what the alternative meant, and almost involuntarily her answer burst from her lips like a cry—"I cannot let you go!"

He knelt at her side and placed his arm round her with infinite tenderness. "You do love me, Kate?" he whispered.

She did not repulse him, neither did she reply, but he took her answer for granted, and pressed his lips to her cheek once more.

"I would have staked my life on your sincerity, my darling," he said. "I felt that there was nothing of the coquette in your nature. I know you too well. Kiss me, Kate."

She did so; his impetuosity had overwhelmed her, carried her away like a flood, and she yielded herself to fate. Why should she not? She had never known anyone towards whom she had felt as she did towards this man. All her occupations and amusements were sweetened by his presence. He shared every taste she had; he was ever in her thoughts, and she was happiest when he was by her side. She honoured and respected him, and saw no other man when he was near. Surely this was love. Such thoughts as these crowded upon each other in her brain as he knelt beside her, but something incomprehensible, something whose existence in her mind she had not known, came between her heart and her lips when she essayed to speak. She had kissed him at his command, and felt she had done him no wrong.

"May I speak to your father, my darling?" he asked. "May I tell him that you love me? May I ask him to give you to me?"

"Yes."

The die was cast. Bairdsley rose to his feet and took her in his arms. "You have given me the happiness of my life, Kate, and I will never forget that you have entrusted me with the happiness of yours. Good-bye, my own Kate, till this evening."

He kissed her again and went away; and she betook herself to her aunt's room, bewildered and dazed by this sudden and passionate wooing, so strangely out of keeping with Bairdsley's usual impassive, calm demeanour.

The Captain made his appearance soon after the ladies had gone out that evening, and was received by Dr. Harrison.

George Bairdsley answered all his questions with straightforwardness and cordiality. His father allowed him a thousand a year, and he had considerable expectations upon his parent's death. He had saved five or six thousand pounds, which he would settle upon Kate when they married, and would make more liberal provision for her when he came into his property, if Dr. Harrison would accept such an arrangement.

The Doctor professed himself quite satisfied, and was pleased with the openness with which Bairdsley met him. He had nothing more to ask, he said, and would only wish him every happiness. He would not detain him any longer, and, he added with a smile, thought that he would find Kate with her aunt at the band. Captain Bairdsley expressed his thanks in a few well-chosen words, and left the bungalow to seek his *fiancée*. Mrs. Tenterdale and Mrs. Brent were with her in the carriage, but after bidding him "good evening" they got out and went away, leaving him alone with Miss Harrison.

The carriage had drawn up some little way from the stand, and the strains of "Queen of my Heart" came to them, softened by distance, as he took his seat at her side.

"I have seen your father, Kate," he began.

"What did he say?" she asked.

"He said 'Yes,' Kate. What do you say?"

The band was playing "Be wise in time," from "Dorothy," and Kate's fan was half unconsciously beating time to the music as she answered "Yes," while the band played on its unheeded warning, "Be wise in time."

*(To be continued.)*

